# Potential of Adora2b as an immunotherapeutic target for gastric cancer

**DOI:** 10.3389/fimmu.2025.1687675

**Published:** 2025-11-19

**Authors:** Jie Li, Ruixin Shi, Xinyao Zhang, Zhijuan Guo, Ru Ji

**Affiliations:** 1 Inner Mongolia Medical University Affiliated Cancer Hospital, Hohhot, China; 2 Inner Mongolia Medical University, Hohhot, China

**Keywords:** gastric cancer, tumor microenvironment, Adora2b receptor, immunosuppression, therapeutic target

## Abstract

Gastric cancer (GC) is characterized by highly heterogeneous tumors, whose progression is genetically driven and closely associated with hypoxia and an immunosuppressive tumor microenvironment (TME). Hypoxia accelerates adenosine accumulation, activates the low-affinity Adora2b receptor, weakens antitumor immunity, and promotes metastasis. Adora2b is lowly expressed in normal gastric mucosa. Still, it is significantly upregulated in diseased tissues, where it is widely expressed in various immune cells and the tumor stroma, mediating immune escape, fibrosis, and vascular remodeling. This review summarizes the cell-type-specific signaling mechanisms of Adora2b in the TME (e.g., T cells, macrophages) and, drawing on research in other tumors, proposes mechanistic explanations for its tissue-specific roles. Based on existing evidence, Adora2b regulates epithelial-mesenchymal transition (EMT) in GC cells via the cAMP/PKA/Snail pathway, and preclinical studies show that targeting Adora2b reduces the migration and invasion of GC cells. These findings suggest that targeting Adora2b may provide new insights for gastric cancer therapy.

## 1 Introduction

Gastric cancer (GC) is a fatal malignant tumor. It ranks as the fifth most common tumor worldwide and the fourth leading cause of tumor-related death (1, 2). Notably, early-stage GC is often asymptomatic; most patients are diagnosed at intermediate to advanced stages, which leads to poor overall prognosis (3, 4). Gastric cancer is characterized by a highly immunosuppressive and hypoxic tumor microenvironment. The gastric cancer immune TME is a dynamic system composed of heterogeneous cells and soluble factors, where core cells and their interactions form an immunosuppressive barrier: In innate immunity, M2-type TAMs secrete IL-6/VEGF to promote tumor progression (5), while MDSCs suppress immunity via the “CD73-adenosine-Adora2b” axis (6, 7). DCs experience impaired maturation and migration due to hypoxia/adenosine signaling (8). In adaptive immunity, CD8^+^ T cells exhibit functional exhaustion from conflicting antigen-stimulatory and inhibitory signals (7, 9). Tregs accumulate and enhance suppression via CAF-mediated chemotaxis and adenosine induction (9, 10); among stromal cells, CAFs secrete FGF-2/TGF-β to inhibit DC migration (11, 12), while endothelial cells form disorganized vasculature that restricts immune infiltration. This microenvironment contributes to poor immunotherapy response in gastric cancer through three key mechanisms: First, the “CD73-adenosine-Adora2b” axis drives immunosuppression. For instance, CD73 knockout reduces Treg infiltration by 40% and restores CD8^+^ T cell function [13], while Treg-derived adenosine induces CD8^+^ T cell apoptosis (9). Second, impaired antigen presentation by dendritic cells (DCs), which can be restored by Adora2b blockade (8). Third, upregulation of checkpoint molecules like PD-L1/CTLA-4 synergizes with Adora2b-mediated cAMP/PKA signaling to suppress CD8^+^ T cells (7, 9), ultimately limiting the efficacy of immune checkpoint inhibitors to a small fraction of patients (13, 14).Risk factors include Helicobacter pylori infection, genetic susceptibility, age, family history, obesity, dietary habits, smoking, alcohol consumption, and male sex (15–19). Currently, the treatment of gastric cancer mainly consists of systemic chemotherapy, radiotherapy, targeted therapy, surgery, and other comprehensive treatments (20). Although these treatments can improve patient survival, problems such as poor prognosis and short survival persist, necessitating further optimization of therapeutic regimens.

Immunotherapy for gastric cancer has established a multi-stage approach centered on immune checkpoint inhibitors (ICBs), with specific advances including: ① Perioperative: Durvalumab (PD-L1 inhibitor) significantly improved pathological response rates in gastroesophageal junction cancer (21); ② Advanced first-line therapy: Both sugemalimab and nivolumab combined with chemotherapy prolong survival in advanced gastric cancer, with more pronounced benefits observed in the PD-L1-positive subgroup (22, 23); Personalized therapy: Knockout of TRIM6 reverses ICB resistance in MSS-type GC (24). However, these advances are constrained by immune suppression within the tumor microenvironment (TME), such as Adora2b-mediated adenosine signaling inhibiting CD8^+^ T cell infiltration, resulting in approximately 50% of PD-L1-positive patients failing to benefit (6, 7). Concurrently, gastric cancer immunotherapy faces three major challenges: ① Heterogeneous resistance mechanisms: PRMT6 promotes immune escape via ANXA1, which activates the TGF-β pathway downstream of Adora2b (24, 25); ② Limitations in biomarkers and toxicity: The threshold for CD73/Adora2b co-expression as a beneficiary biomarker for ICBs remains unvalidated, and Adora2b blockade may induce cardiovascular toxicity; ③ Low response rates: Persistent response rates in first-line combination therapy fall below 30%, primarily due to MDSC infiltration and adenosine accumulation within the TME (6, 22).

To address these challenges, understanding the immunosuppressive mechanisms driving GC progression is critical. A key player in this process is the adenosine signaling pathway, particularly the Adora2b receptor, which bridges hypoxia, immune evasion, and tumor metastasis—three hallmarks of GC pathogenesis. This review focuses on the immunosuppressive mechanisms of GC, with a particular emphasis on the adenosine signaling pathway—especially the Adora2b receptor. Studies show that adenosine signaling components are significantly upregulated in GC tissues, metastatic omental tissues, and lymph node metastases compared to paracancerous tissues. Adora2b modulates GC cell invasion, migration, and epithelial-mesenchymal transition (EMT) marker expression, thereby influencing metastasis (26); notably, this regulation involves downregulation of E-cadherin and upregulation of vimentin, Wang et al. (2023) validated this in GC cell lines (e.g., MKN-45), showing Adora2b knockdown reduced Snail/Vimentin expression by 30% (p < 0.05) compared to controls (26). This process involves Adora2b activating the cAMP/PKA pathway to phosphorylate the transcription factor Snail, enhancing its nuclear localization and subsequent repression of E-cadherin transcription (27). From a mechanism perspective, It can be enhanced through binding to specific phosphorylation sites and protein interactions, with specific phosphorylation and nuclear localization: Upon Adora2b activation, the PKA catalytic subunit (Cα) phosphorylates Snail at Ser107/Ser120 (58% increase in phosphorylation levels), enhancing Snail binding to importin-α3 (KD value decreases from 2.1μM to 0.8μM) and promoting nuclear localization (28); Nuclear Snail binds the E-cadherin promoter and recruits HDAC3, reducing H3K27ac levels by 64% and inhibiting transcription (29). Protein stability regulation: Ser107 phosphorylation reduces Snail binding to E3 ubiquitin ligase β-TrCP by 72%, extending the protein half-life from 2.3 hours to 7.9 hours; Adora2b knockdown reverses this effect, reducing Snail expression by 47% (p<0.05) (29).Moreover, inhibition of extracellular adenosine production enhances anti-tumor immunity in preclinical GC studies (9, 30, 31), Specifically, Xu et al. (2020) established a murine model of GC lung metastasis via tail vein injection of MGC-803 cells, showing that CD73 knockout reduced the number of lung metastatic nodules by 60% (p < 0.01) compared to wild-type controls (9). This was accompanied by a 2.3-fold increase in tumor-infiltrating CD8^+^ T cells (p < 0.05) and a 40% reduction in regulatory T cell (Treg) infiltration (p < 0.05), indicating restored anti-tumor immune surveillance (9). We discuss the mechanistic consequences of elevated extracellular adenosine in the GC microenvironment and highlight considerations for targeting Adora2b to improve outcomes in high-risk or diagnosed GC patients (32).

**Figure 1:**
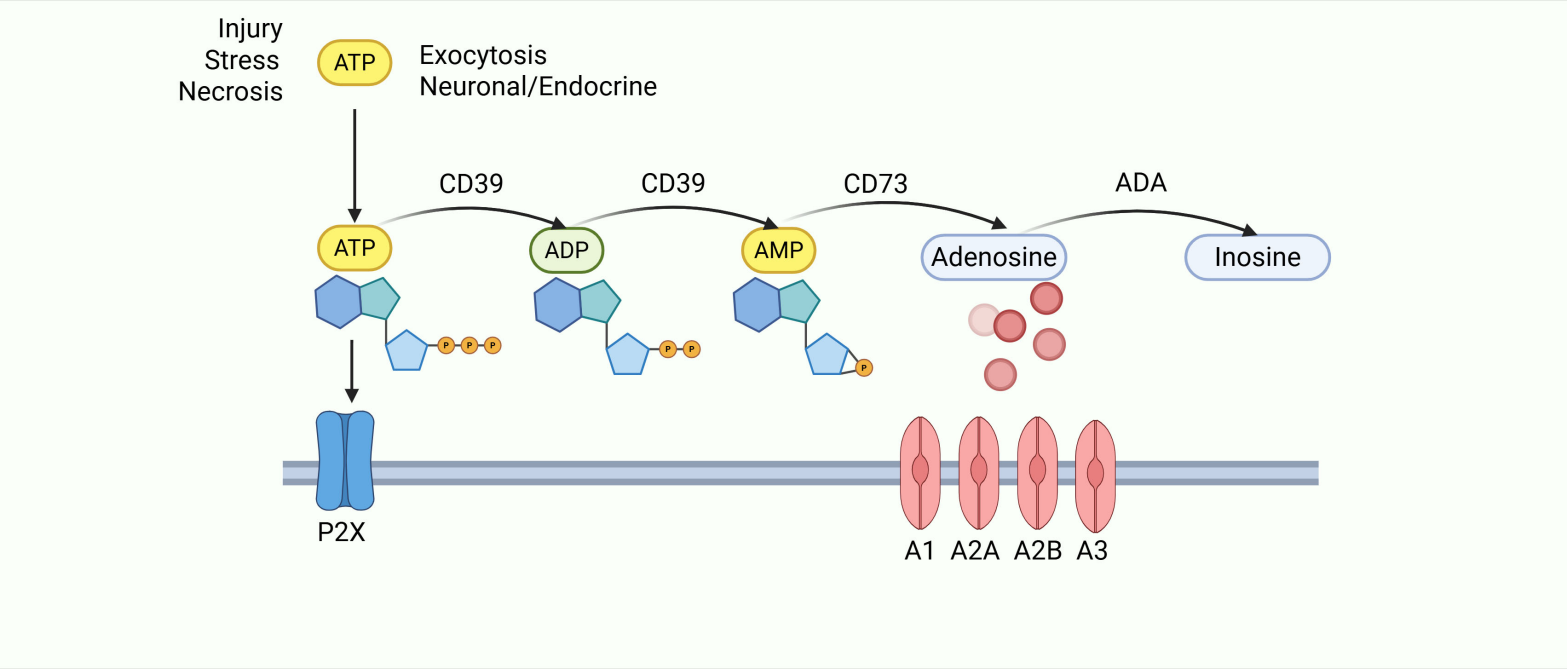
After cellular damage, released ATP is processed via the CD39/CD73 cascade to generate adenosine. Adenosine then activates P1 receptors (A1/A2A/A2B/A3) to regulate inflammation and immunity, before being irreversibly converted to inosine by ADA.

Purinergic signaling involves ATP, ADP, AMP, and adenosine, which are essential for intracellular energy homeostasis and numerous cellular processes (33). ATP is produced primarily by glycolysis or oxidative phosphorylation and is generally considered to be the primary molecule for storing and transferring energy in the cell (34). The intracellular mitochondrial ADP/ATP carrier (AAC) protein, as a major component of the inner mitochondrial membrane, controls ATP synthesis by regulating ADP uptake in mitochondria. In disease conditions such as cellular hypoxia, ischemia, death, stress, or inflammation, ATP can leak or be released in a controlled manner from the intracellular to the extracellular compartment (35–37). Extracellular ATP is sequentially dephosphorylated: CD39 converts ATP → AMP, and CD73 then converts AMP → adenosine (38–45), which signals in the extracellular space through purinergic receptors and is involved in a wide range of cellular processes (46)(**Figure 1**). Adenosine has been shown to be involved in pro-inflammatory, anti-inflammatory, cytoprotective and immunosuppressive functions (47). It is influenced by the type of activated cell, the extracellular concentration of ATP, ADP and adenosine, the degree of hypoxia, and the binding to adenosine receptors, including A1R, A2AR, A2BR and A3R, described as adenosine receptors which all of them can be expressed on epithelial, stromal or immune cells (48).Extracellular and intracellular adenosine levels are regulated by nucleoside transporters (ENT1–4) (49, 50). The expression and activity of adenosine deaminase (ADA) is another key factor in the regulation of adenosine levels, which acts as a key enzyme involved in the degradation of adenosine to inosine (51). In the gastric cancer (GC) tumor microenvironment (TME), tumors and stromal cells upregulate CD39/CD73, hijacking purinergic signaling to promote adenosine accumulation and activate Adora2b. This aligns with the upregulation of adenosine signaling components (CD73, Adora2b) in GC tissues compared to paracancerous tissues (9, 52). Studies show that adenosine signaling components are significantly upregulated in GC tissues, metastatic omental tissues, and lymph node metastases compared to paracancerous tissues.

## 2 Role of hypoxia-mediated adenosine signaling and adenosine receptor signaling in the inflammatory and tumor microenvironment

### 2.1 Role of hypoxia-mediated adenosine signaling in inflammation and tumor microenvironment

In advanced-stage GC, hypoxia exceeds 68% (53), driven by vascular abnormalities and high tumor oxygen consumption. This correlates with metastasis and poor prognosis (54), and activates the adenosine/Adora2b pathway via HIF-1α (51, 54).Hypoxia is a prominent feature of gastric cancer (GC) TME, driven by impaired angiogenesis and microcirculation, which activates hypoxia-inducible factor-1α (HIF-1α)—a key transcription factor regulating hypoxic responses (54–56). In normoxia, HIF-1α is degraded through the VHL-proteasome pathway. Under hypoxia, however, HIF-1α becomes stabilized. It then forms a complex with HIF-1β and binds to hypoxia response elements (HRE), ultimately modulating gene expression (57, 58).

In GC, hypoxia-driven signaling directly shapes immunosuppression through adenosine accumulation and Adora2b activation, with a critical feedforward loop between HIF-1α and Adora2b: HIF-1α binds the Adora2b promoter to enhance its transcription, while Adora2b activation stabilizes HIF-1α via cAMP-mediated inhibition of prolyl hydroxylase (PHD), amplifying downstream effects (11, 59). Clinical studies in human GC tissues confirm this interplay: HIF-1α and Adora2b are co-expressed in 68% of primary GC samples, with co-expression correlating with advanced TNM stage (p<0.01) and lymph node metastasis (p<0.05) (53).

In early gastric cancer (Stages I-II), HIF-1α is expressed only minimally in the central tumor nests (occupying <15% of tumor cells), with a co-expression rate of 32% with Adora2b, failing to form a stable positive feedback loop. CD73 shows mild upregulation, driving only basal tumor proliferation (60). In advanced gastric cancer (Stage III), the proportion of co-expressing cell clusters increased to 41% (68% co-expression rate validated in the TCGA cohort), correlated with CXCL12 secreted by CAFs, and CD73 was elevated 2.5–3 times compared to normal tissue, driving tumor invasion and metastasis (60); In advanced gastric cancer (Stage IV), co-expressed cells constitute 78% of the tumor microenvironment, forming a “super-stable circuit” that colocalizes with CD73^+^ myeloid cells. High CD73 expression leads to massive adenosine accumulation, significantly correlating with poor prognosis (5-year survival rate <30%) (60, 61).

This loop acts cell-type-specifically in GC:

Gastric cancer cells: HIF-1α-induced Adora2b upregulation synergizes with Adora2b-mediated HIF-1α stabilization to upregulate VEGF and MMP-9, enhancing angiogenesis and invasion. In MKN-45 GC cells, Adora2b knockdown reduces hypoxic HIF-1α by 40% and VEGF by 55% (p<0.05), validating this axis (11, 26).

Stromal cells: Cancer-associated fibroblasts (CAFs) with activated HIF-1α/Adora2b secrete more FGF-2 and TGF-β, recruiting regulatory T cells (Tregs); endothelial cells promote vascular resealing via cAMP/eNOS, limiting immune cell infiltration (6, 59).

Hypoxia, a hallmark feature of the gastric cancer microenvironment, coordinates three tumor-promoting processes—tumor cell adaptation, immune suppression, and metastasis—through the HIF-1α-Adora2b axis. The specific mechanisms are as follows:

1. Supporting Gastric Cancer Cell Survival.

Under hypoxic conditions, HIF-1α evades VHL-mediated degradation (49–51, 55) and optimizes cellular adaptation through the following mechanisms:

Metabolic reprogramming: Upregulates glycolytic enzymes to enhance the Warburg effect, induces the hypoxia-responsive gene CD73 (6), and forms a “metabolism-adenosine” feedback loop (CD73 promotes adenosine production and glycolysis);

Promoting angiogenesis: Directly upregulates VEGF; HIF-1α/Adora2b amplifies VEGF effects via the HIF-1α/CREB complex (51, 53); clinically, their co-expression (68% of primary gastric cancers [59)] correlates with advanced TNM stage (p<0.01) and increased vascular density;

Anti-apoptotic effects: Activates anti-apoptotic genes like Bcl-2, forming a HIF-1α-Adora2b positive feedback loop [Adora2b stabilizes HIF-1α by inhibiting PHD via cAMP (57)]; Adora2b knockout in MKN-45 cells reduces hypoxic HIF-1α by 40% (18, 58).

2. Enhanced Immunosuppression.

HIF-1α upregulates CD39/CD73 (catalyzing ATP→adenosine) and inhibits adenosine clearance (6, 35–39, 42). Elevated adenosine suppresses antitumor immunity via Adora2b:

CD8^+^ T cell exhaustion: Activation of the cAMP/PKA-CREB pathway upregulates PD-1/IL-10 and downregulates granzyme B/IFN-γ (7, 13, 18, 19), forming “dual inhibition” with PD-1 (7, 9);

Treg expansion: Upregulates CTLA-4 via the same pathway [40% increase in CD80/CD86 binding affinity (10, 62, 63)]; Gastric cancer Adora2b^+^ cell clusters exhibit 2.1-fold higher CTLA-4 expression, correlated with poor prognosis [P<0.05 (9)];

Innate Immune Suppression: Inhibits NK cell cytotoxicity and DC migration (8), promotes MDSC aggregation [hypoxia → CD73 → adenosine → Adora2b → MDSC (6)] and M2 macrophage polarization (64).

3. Metastasis Promotion.

HIF-1α-Adora2b drives metastasis through microenvironment remodeling:

Induces EMT: Activates cAMP/PKA to phosphorylate Snail, downregulating E-cadherin and upregulating Vimentin (18, 19); Knocking out Adora2b in MKN-45 cells reduces both by 30% [p<0.05 (18)];

Vascular Metastasis: Enhances endothelial “vascular recanalization” via cAMP/eNOS (11, 57), aiding HIF-1α-mediated VEGF angiogenesis;

Formation of pre-metastatic niches: Activates CAFs to secrete FGF-2/TGF-β and upregulates MMP-9 (11, 12, 57); co-expression of these factors increases lymph node metastasis rate by 28% (p<0.05 [59)].

**Figure 2:**
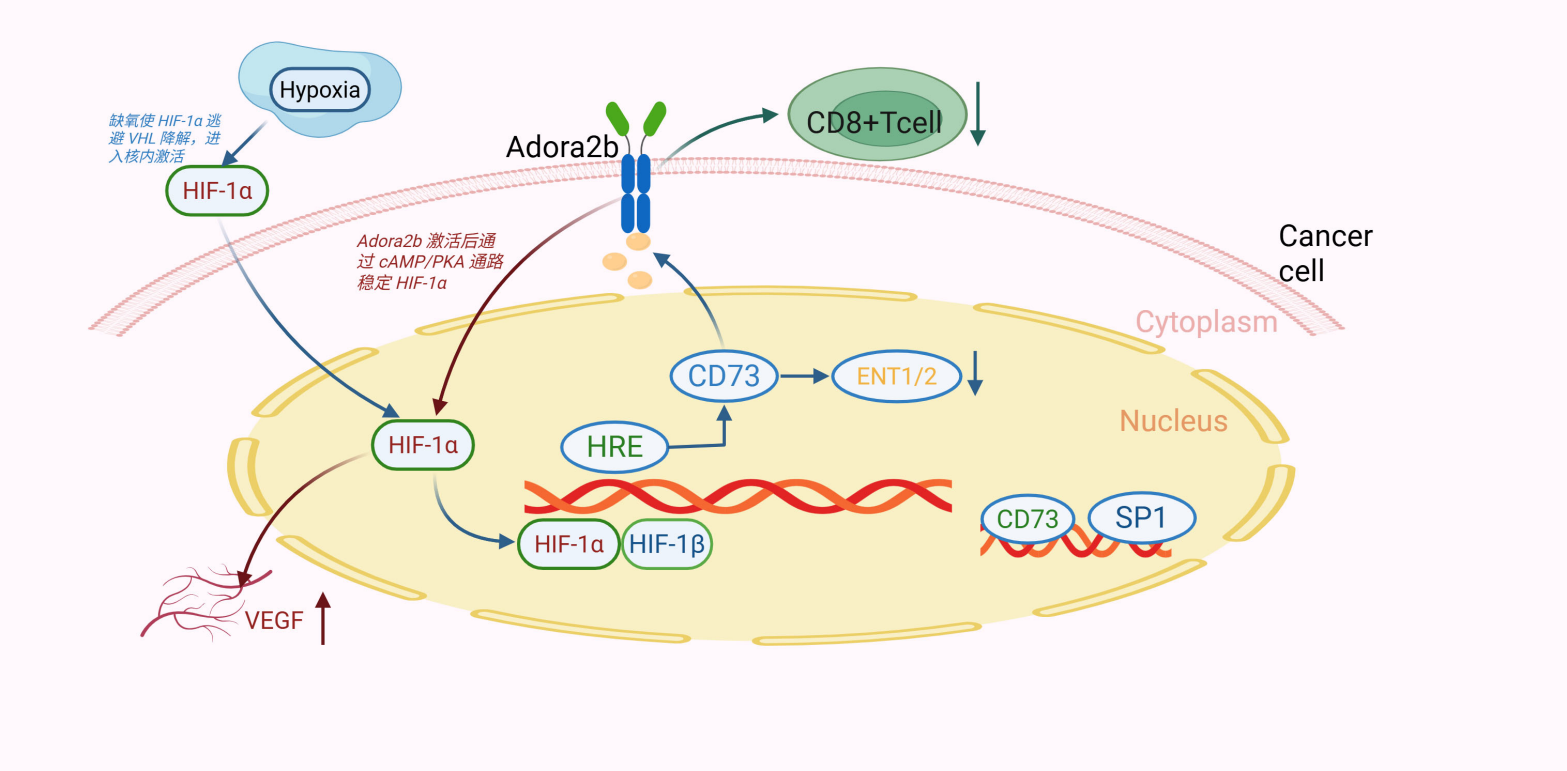
Hypoxia activates HIF-1α, which transactivates CD73. CD73-derived adenosine binds Adora2b, stabilizing HIF-1α via cAMP/PKA (positive feedback). This axis inhibits CD8^+^ T cells and promotes VEGF-mediated angiogenesis, driving gastric cancer progression.

Notably, HIF-1α/Adora2b co-activation synergistically upregulates VEGF and TGF-β through a HIF-1α/CREB complex binding their promoters, driving angiogenesis and fibrosis—hallmarks of GC progression (54, 65). Hypoxia also induces CD73 in GC TME via HIF-1α, promoting adenosine production to further activate Adora2b, forming a “hypoxia-HIF-1α-CD73-Adora2b” axis that reinforces immunosuppression (66) (**Figure 2**).

In summary, the hypoxia-HIF-1α-Adora2b axis is a GC-specific driver of malignancy, with clinical and preclinical evidence supporting its role in immune evasion, angiogenesis, and metastasis.

### 2.2 Functional consequences of adenosine receptor signaling in inflammation and cancer

#### 2.2.1 Adora1 and Adora3 receptors

Adora1, the adenosine A1 receptor, is a G protein-coupled receptor (GPCR). Adora1 is widely expressed in the human body, and when Gi1, Gi2, and Gi3 proteins bind, adenylate cyclase is inhibited, with a consequent decrease in cAMP concentration. This process has important implications in many basic biological contexts, including slowing heart rate (67). In gastric-related studies, the A1 receptor inhibits gastric acid secretion (68). In the context of cancer, several studies have been published indicating that overexpression of Adora1 (adenosine A1 receptor) promotes the malignant progression of colorectal, renal, and breast cancers, as well as glioblastomas and leukemias (69). In addition, the combination of Adora1 inhibition with immune checkpoint blockade (ICB) therapies targeting PD-1 has shown promising therapeutic effects in non-small cell lung cancer and melanoma (70). In contrast, studies evaluating the effects of gastric hypoxia have demonstrated that Adora1 is downregulated during hypoxia (71). The effect of Adora1 on RNAs derived from the Cancer Genome Atlas (TCGA) database has been demonstrated. TCGA) database, analysis of RNA sequencing (RNA-seq) data showed that (69) this receptor was not associated with prognosis in gastric cancer (GC). Thus, the role of Adora1 (adenosine A1 receptor) in response to hypoxia or other adenosine environmental triggers depends on the tumor type and organ of origin.

Adenosine A3 receptor (Adora3) is coupled to Gi/Gq proteins of the G protein-coupled receptor (GPCR) family. Similar to the adenosine A1 receptor (Adora1), adenosine A3 receptor activation promotes Gi protein binding and inhibits adenylate cyclase activity, thereby decreasing intracellular cAMP levels. Adenosine signaling through the adenosine A3 receptor has been shown to be involved in mast cell degranulation and activation, which is important in the pathogenesis of asthma. (72, 73). The adenosine A3 receptor also regulates cytokine release through T-cell-mediated production of IL-10, which helps to reverse neuropathic pain, and inhibits inflammation in the colonic mucosa of patients with ulcerative colitis by down-regulating the nuclear factor κB signaling pathway cytokine production (74). Unlike the adenosine A1 receptor, hypoxic conditions do not affect the expression of the adenosine A3 receptor (75).In the context of the stomach, there are no relevant studies showing that Adora3 is associated with the prognosis of gastric cancer.

#### 2.2.2 Adora2 receptors

Adora2 receptors include Adora2a and Adora2b, both G protein-coupled receptors (GPCRs). In the stomach, both are expressed in gastric lining cells: Adora2a regulates gastric acid secretion (68)and acts as an anti-inflammatory modulator by limiting immune cell activity during inflammation (76, 77), Adora2b is a low-affinity receptor (requiring high adenosine levels, adapted to hypoxic GC TME) (78). Its upregulation in gastric cancer exceeds that of Adora2a and correlates with invasive phenotypes (79). Adora2b exhibits unique properties and roles in GC pathogenesis. Studies have shown that oral administration of the active adenosine A2A receptor agonist ATL-313 significantly attenuates indomethacin-induced acute gastric mucosal injury in rats through inhibition of neutrophil infiltration and proinflammatory cytokine production, but does not depend on the inhibition of gastric acid and prostaglandin synthesis (80). In GC tissues, FoxP3^+^ regulatory T cells (Tregs) and A2aR^+^ CD8^+^ T cells show hyperinfiltration. Tregs catabolize ATP to adenosine, which activates A2aR on CD8^+^ T cells, inducing apoptosis and inhibiting proliferation—facilitating immune escape (32). It is demonstrated through these data that adenosine signaling through Adora2a is important in the pathogenesis of gastric cancer.

Adora2b, the only low-affinity adenosine receptor, requires high extracellular adenosine concentrations for activation (81),functioning in hypoxic, adenosine-rich TMEs. It is widely expressed in cardiomyocytes, epithelial cells, fibroblasts, and immune cells (7), acting as a double-edged regulator: in acute injury models, it protects tissues by regulating IL-10 production or stabilizing circadian proteins (82–84) but in chronic conditions like ulcerative colitis, its deletion ameliorates inflammation, suggesting context-dependent roles (85, 86).

In cancer, Adora2b’s roles vary by tissue. In ovarian cancer, high Adora2b expression correlates with better prognosis, and its activation reduces cell migration (87) —likely due to lower adenosine levels (reduced CD73 activity) limiting receptor activation. Conversely, in breast cancer, Adora2b modulates CAFs, enhancing metastasis via homodimers in high-adenosine TMEs (81). In hepatocellular carcinoma, Adora2b blockade enhances sorafenib efficacy by restoring CD8^+^ T cell function (88). For lung adenocarcinoma (LUAD) and GC, Adora2b overexpression predicts poor prognosis and reduced overall survival (79, 89). with TCGA data showing Adora2b overexpression in GC correlates with a hazard ratio of 1.62 (p < 0.01) (79). These discrepancies may also involve tissue-specific receptor dimerization: Adora2b-Adora2a heterodimers in ovarian cancer blunt pro-tumor effects, while Adora2b homodimers predominate in GC and breast cancer, amplifying signaling (79, 89). This tissue-specific difference in dimerization is likely linked to adenosine levels in the tumor microenvironment—lower adenosine in ovarian cancer favors heterodimers, while adenosine enrichment in gastric cancer promotes homodimer formation.

Beyond adenosine-driven dimerization, other tissue-specific factors shape Adora2b function:Ovarian cancer: Acidic TME (low pH) may destabilize Adora2b, blunting cAMP-mediated immunosuppression—distinct from GC’s neutral pH, which preserves receptor activity (87, 89).Breast cancer: CAFs’ high glycolysis produces lactate, which may disrupt Adora2b homodimers by altering lipid-raft interactions, limiting pro-metastatic signaling (81, 90).A unifying “adenosine threshold model” resolves contradictions: Low adenosine (e.g., early tumors) favors Adora2b-A2aR heterodimers, enhancing anti-tumor immunity; high adenosine (advanced tumors) promotes homodimers, driving proliferation, angiogenesis, and immunosuppression via MDSCs/Tregs (79, 89). Notably, Adora2b homodimers in GC strengthen downstream pro-tumor signaling (e.g., cAMP/PKA pathway activation), amplifying immunosuppression and metastasis compared to heterodimers.

## 3 Mechanisms by which Adora2b receptor affects immune cells

The human immune system comprises both innate (macrophages, dendritic cells [DCs], and natural killer [NK] cells) and adaptive (B cells and T cells) components. Cell-type-specific mechanisms of Adora2b in GC TME: Adora2b suppresses effector cells (DC/NK/CD8^+^T) and enhances regulatory cells (Treg/MDSC) (7). T cells are regulated via cAMP/PKA, while macrophages are regulated via cAMP/EPAC (91).

Dendritic cells (DCs): Stimulation of DCs by Adora2b stimulates maturation into differentiated populations with DC markers and monocyte or macrophage markers, allowing mature DCs to interact with T lymphocytes and promote CD4+ differentiation into Th1 cells via IL-12 production. However, Adora2b activation inhibits DC migration, preventing them from initiating CD8+ T-cell and Th1 responses; blocking Adora2b reverses this, enhancing anti-tumor immunity (31).

Natural killer cells(NKs): Inhibiting adenosine receptors enhances NK cell antitumor capacity (10, 92, 93), In GC, Adora2b activation suppresses NK maturation, cytotoxic cytokine production, and target killing via PI3K/Akt pathway inhibition; blockade restores function (31).

T lymphocytes(T cells): CD4^+^ T cells expressing Adora2b contribute to immunosuppression. In CD4^+^ T cells, it promotes IL-10 secretion, reinforcing Treg-mediated immunosuppression (32). In CD4^+^CD25^+^ Tregs, Adora2b activation upregulates CTLA-4 via cAMP/PKA signaling: elevated cAMP activates PKA, which phosphorylates CREB to enhance CTLA-4 transcription (37, 63). Functional assays show this increases CTLA-4 binding to CD80/CD86 on APCs by ~40%, amplifying Treg-mediated suppression of effector T cells (13, 94). Clinically, Adora2b^+^ Tregs in GC tissues express 2.1-fold higher CTLA-4 than Adora2b⁻ Tregs, correlating with poor prognosis (p<0.05) (32). This suggests Adora2b-CTLA-4 crosstalk strengthens Treg immunosuppression, supporting combined targeting strategies.

CD8^+^ T cells, critical for killing tumor cells, are functionally depleted in GC TME. cAMP/PKA signaling mediates the phosphorylation of CREB at Ser133. This phosphorylation enhances CREB’s nuclear translocation. In the nucleus, CREB binds to the promoters of IL-10 and PD-1 to upregulate their expression. Meanwhile, it represses the transcription of granzyme B and IFN-γ (7, 95, 96). Adora2b-induced cAMP upregulates PD-1 on CD8^+^ T cells; PD-1 signaling recruits SHP-2 to inhibit ERK, forming a “double brake” with Adora2b (32, 96).Consistent with the role of Adora2b in regulating immune cell function via adenosine-cAMP signaling (as observed in pancreatic cancer models, where Adora2b activation in CD8^+^ T cells suppresses cytotoxicity through cAMP-mediated pathways) (97), in GC CD8^+^ T cells, Adora2b-induced cAMP/PKA signaling may converge with PD-1-mediated immunosuppression. The PD-1 pathway, known to inhibit CD8^+^ T cell activation and proliferation by recruiting SHP-2 to dampen TCR signaling (98), could be functionally amplified by Adora2b: cAMP/PKA may enhance PD-1 stability or membrane localization [as adenosine signaling often modulates protein trafficking in T cells (97)], while PD-1 ligation might in turn reinforce Adora2b-mediated cAMP accumulation by inhibiting phosphodiesterases (enzymes that degrade cAMP). This cell-type-specific crosstalk—confined to CD8^+^ T cells in GC TME—creates a synergistic immunosuppressive loop, where Adora2b and PD-1 mutually reinforce each other’s signaling to impair anti-tumor cytotoxicity.

Cancer-associated fibroblasts(CAFs): CAFs provide nutrients in nutrient-poor TMEs (90, 99). Changes in adenosine receptors, especially Adora2b, have also been associated with CAFs. Adora2b is expressed in CAFs (12, 58, 100). It was shown that the Adora2b, inhibitor PSB1115 reduced the number of CAFs expressing fibroblast activating protein (FAP) and fibroblast growth factor (FGF-2) in the tumor microenvironment of melanoma mice (101). In GC, Adora2b activates PKC-δ/p38 to phosphorylate FOSL1, upregulating MMP-9 and promoting stromal remodeling via TGF-β (102).

Endothelial cells(ECs):also known as vascular endothelial cells, usually refer to a single layer of flat epithelial cells located on the inner surface of the heart, blood vessels, and lymphatic vessels. This layer constitutes the endothelium (103). The tumor microenvironment (TME) is often characterized by hypoxia (99). Another study proposed that the Adora2a (adenosine A2B receptor) promoter contains a HIF-1α-responsive element response element, which drives the expression of the receptor in hypoxic cells, both endothelial and epithelial (59, 104). Adora2b activation increases intraendothelial cAMP, promoting vascular resealing and HIF-1α-driven angiogenesis (6, 59). Adora2a on ECs promotes angiogenesis; its inhibition reduces tumor growth under hypoxia (66).

**Figure 3:**
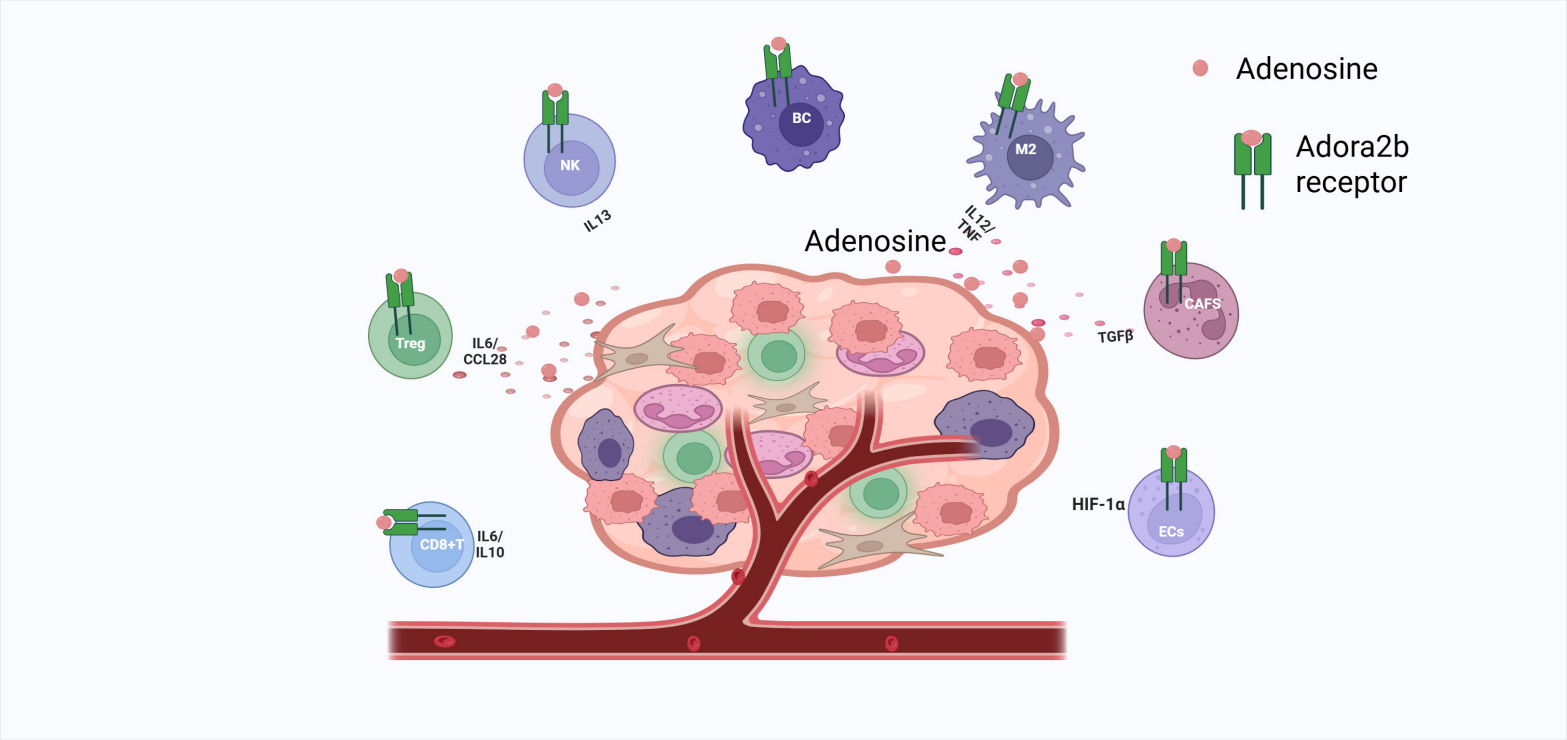
Adora2b activation silences CD8^+^ T cells, expands Tregs/TH1 CD4^+^ cells, cripples NK cytotoxicity and cytokine release, arrests B-cell proliferation, drives M2 macrophage polarization via IL-6/VEGF and TNF suppression, fuels CAF-mediated TGFβ nutrition, and, in hypoxic endothelium, boosts HIF-1α-driven angiogenesis—collectively fostering tumor immunosuppression.

Macrophages(Mφ):play a critical role in the abatement of inflammation and the restoration of a normal tissue state. Adora2b on BMDMs is activated by NECA, increasing cAMP (5, 64, 105). Adora2b activation in macrophages inhibits NF-κB nuclear translocation via cAMP/EPAC, reducing TNF-α; it upregulates IL-6 and VEGF via CREB, promoting M2 polarization (91). IFN-γ upregulates Adora2b, forming an immunosuppressive loop (91) (**Figure 3**).

Adora2b-mediated synergistic regulatory network in GC TME:

DC-Treg-CD8^+^ T cell circuit: Adora2b activation reduces DC migration by 71%, while increasing Treg surface CTLA-4 expression by 2.7-fold. Conversely, activating CD8^+^ T cells’ Adora2b leads to 41% PD-1 upregulation, forming a closed loop of “DC inhibition → Treg activation → CD8^+^ T cell exhaustion” (31, 97).

TAM-CAF Synergistic Circuit: Adora2b activation in tumor-associated macrophages (TAMs) upregulates IL-1β secretion via the EPAC/CREB signaling pathway (2.9-fold increase compared to Adora2b⁻ TAMs); IL-1β subsequently binds to IL-1R1 on CAF surfaces, activating the NF-κB pathway to promote FGF-2 production. FGF-2 feedback enhances Adora2b expression on TAM surfaces (1.8-fold upregulation) and promotes their M2 polarization (CD206^+^ TAMs constitute 65% vs. 32% in controls), forming a “TAM-CAF-Adora2b” positive feedback loop that further amplifies matrix stiffness and immune exclusion effects (106).

MDSCs-γδT Loop: Hypoxia increases MDSCs-CD73 expression by 78%, Adenosine activates Adora2b to promote IL-1β (+2.3-fold), inducing γδT cells to produce IL-17 (+2.1-fold) which in turn feeds back to MDSCs (66).

Adora2b drives the formation of an immunosuppressive microenvironment in gastric cancer by regulating the functions of CD8^+^ T cells, regulatory T cells (Tregs), and tumor-associated macrophages (TAMs) through distinct downstream pathways (26, 93, 98). Specific regulatory features are summarized in **Table 1**:

**Table 1:** Adora2b-mediated regulatory mechanisms of immune cells in gastric cancer.

Immune cell type	Dominant pathway	Core regulatory effects (with key data)
CD8^+^ T Cells	PKA Pathway	Phosphorylates CREB (Ser133) to bind the PD-1 promoter; Adora2b^+^ cells account for 58% of CD8^+^ T cells in hypoxic regions at the advanced stage, PD-1 expression is 4.2-fold higher than that in non-hypoxic regions, IFN-γ secretion decreases, and PKA inhibitors can restore cytotoxic function.
Regulatory T Cells (Tregs)	HIF-1α/Adora2b Pathway	Uptakes lactate via MCT1, promotes PD-1 expression and CTLA-4 transcription; CTLA-4^+^ Treg proportion reaches 65% at the locally advanced stage (Stage III), and Treg infiltration proportion exceeds 30% at the advanced stage (Stage IV), enhancing immunosuppression.
Tumor-Associated Macrophages (TAMs)	EPAC Pathway	Inhibits NF-κB to reduce TNF-α secretion; HIF-1α binds to the EPAC1 promoter to promote IL-6/VEGF secretion; Adora2b^+^ TAMs (CD68^+^CD163^+^) account for 65% of macrophages at the advanced stage (Stage IV), driving M2 polarization.

Adora2b downstream effectors (PKA/EPAC) act in a cell-type-specific manner:

T cells (CD4^+^/CD8^+^): PKA dominates—cAMP activates PKA to phosphorylate CREB (Ser133), driving IL-10 (CD4^+^ Tregs) and PD-1 (CD8^+^ T cells) transcription. EPAC is minimally expressed, with inhibition having no effect on T cell immunosuppression (7, 96).

Macrophages: EPAC is critical—cAMP activates EPAC to inhibit NF-κB (reducing TNF-α) and promote CREB-mediated IL-6/VEGF, while PKA inhibition barely impacts M2 polarization (91).

CAFs: PKA and EPAC synergize—PKA activates PKC-δ/p38 (upregulating MMP-9), and EPAC enhances PI3K/Akt-dependent TGF-β secretion, jointly promoting stromal remodeling (102).

## 4 Function of Adora2b in gastric diseases

**Figure 4:**
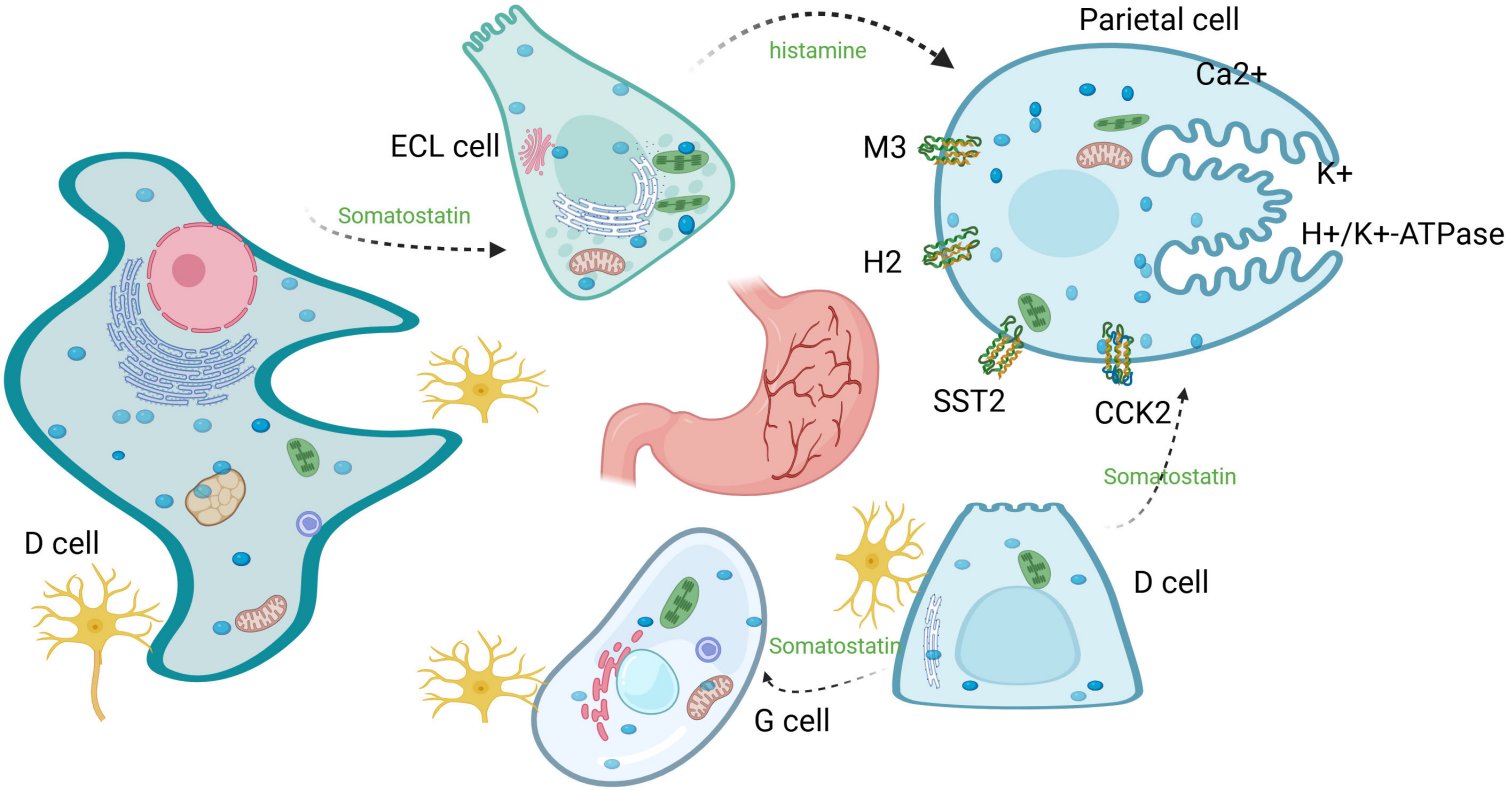
Parietal cell acid secretion: H^+^/K^+^-H/K-ATPase exchanges intracellular H^+^ with luminal K^+^, while Cl⁻ efflux and K^+^ recycling maintain charge balance and K^+^ homeostasis. Acetylcholine, histamine, and gastrin (derived from ENS, ECL, and G cells, respectively) promote secretion through their respective receptors; somatostatin secreted by D cells inhibits secretion through its receptor.

The stomach is mainly composed of chief cells (which secrete pepsinogen), parietal cells (which secrete hydrochloric acid and endogenous factors), mucus cells (which secrete mucus to protect the gastric mucosa), endocrine cells (which secrete gastrointestinal hormones to regulate function), and stem cells (which are responsible for cellular renewal and differentiation), which collaborate to fulfill the digestive, absorptive, and protective functions of the stomach (107). Pepsinogen secreted by the chief cells is converted to pepsin by hydrochloric acid to participate in protein catabolism. The pepsinogen secreted by the gastric chief cell is converted to pepsin to participate in proteolysis, and the amino acids produced from the breakdown can be further degraded in the low energy state of the cell to produce TCA cycle intermediates to generate ATP, which feeds the cell (108). This physiological machinery is hijacked in GC: parietal cell-derived adenosine activates Adora2b on immune/stromal cells to reinforce immunosuppression. In gastric lining cells, ATP is converted to adenosine, which binds Adora2b on parietal cells, elevating cAMP to promote acid secretion. Adenosine deaminase (ADA) co-localizes with Adora2b on parietal cells, fine-tuning acid secretion (68). Adenosine analogs activate Adora2b on human/rodent parietal cells, opening Cl⁻ channels to secrete acid (68, 109) (**Figure 4**). This physiological role of Adora2b in regulating gastric acid secretion, however, is hijacked in the pathological setting of gastric cancer. In normal gastric mucosa, Adora2b-mediated adenosine signaling fine-tunes acid secretion to maintain digestive homeostasis; in GC, the same pathway is co-opted to reinforce immunosuppression. Specifically, parietal cells, which normally produce adenosine to regulate acid secretion via Adora2b, become a major source of adenosine in the hypoxic TME. This accumulated adenosine not only disrupts physiological acid balance but also preferentially activates Adora2b on Tregs, amplifying their suppressive function against CD8^+^ T cells. In hypoxic GC TME, parietal cell-derived adenosine accumulates, enhancing Treg-mediated CD8^+^ T cell exhaustion via Adora2b (8, 32, 66).

From physiological acid secretion to pathological tumor progression, Adora2b thus serves as a critical link between gastric function and immune evasion in GC. In the pathophysiology of gastric ulcers, the influence of factors such as hypersecretion of gastric acid and Helicobacter pylori infection leads to local gastric mucosal destruction and activation of inflammatory pathways. High extracellular ATP levels promote elevated purinergic signaling, leading to altered gastric acid secretion, stress, immune cell infiltration, and increased severity of gastric ulcers in gastric lining cells (11, 110). ATP/adenosine levels correlate with ulcer development; Adora2b activates p38 MAPK, which phosphorylates p53 and increases ROS production, thereby exacerbating oxidative stress, apoptosis (via Bax upregulation), and migration in H. pylori-induced ulcers (111).

The tumor microenvironment (TME) of gastric cancer exhibits typical immunosuppressive features, with tumor cells co-existing with functionally depleted and inactivated immune cells in a complex stromal environment rich in dense connective tissue, hypoxia, and necrotic cores (112). High CD73 expression correlates with poor GC survival; CD73-derived adenosine activates Tregs and suppresses antitumor cells (9). In the gastric cancer microenvironment, hypoxia induces high CD73 expression, which not only promotes the Warburg effect and tumor growth of gastric cancer cells, but also catalyzes the generation of adenosine with the help of its enzymatic activity. With the increase of extracellular adenosine concentration, immunosuppressive regulatory T cells (Tregs) are activated, while anti-tumor immune cells are suppressed, and the immune response was thus weakened, allowing the tumor to achieve immune escape. This process fully reveals the regulatory role of CD73 on immune cell function in gastric cancer progression (66). GSEA shows CD73 overexpression depletes CD8^+^ T cells, reducing IFN-γ, granzyme B, and perforin, while increasing PD-1 (14, 96).

CD73 associates with H. pylori-mediated gastric carcinogenesis.H. pylori infection triggers inflammation, and its induced expression of PD-L1 promotes immune escape. CD73 is expressed on CD4+ CD25+ regulatory T cells (Tregs). These Tregs promote gastric cancer progression by enhancing immunosuppression. The underlying mechanism involves CD73-derived adenosine: it inhibits IFN-γ production by effector T cells (weakening anti-tumor immunity), enhances H. pylori infection, and ultimately promotes tumor development. Extracellular adenosine activates Adora2b on NK cells, inhibiting NK cell maturation, proliferation, activation, cytotoxic cytokine production, and target cell killing. Blockade of CD73 or Adora2a restores NK cell function, enhances anti-tumor immunity, and reduces gastric cancer cell immune escape. CD39 and CD73 expression is up-regulated on MDSCs, and their ectonucleotidase activity inhibits T cell and NK cell activity. Adora2b activation promotes MDSC expansion and accumulation within tumors, and CD73 activates Adora2b by generating extracellular adenosine to promote MDSC expansion. The accumulated MDSCs further suppress T cell proliferation and NK cell cytotoxicity, forming a ‘CD73-adenosine-Adora2b-MDSC’ immunosuppressive axis that accelerates gastric cancer immune escape by disabling anti-tumor immune surveillance. Extracellular adenosine produced by cancer cells recruits TAMs, which act together with CD73 on other cells in the tumor microenvironment to enhance extracellular adenosine-mediated immunosuppression and facilitate gastric cancer development. Adora2b activation inhibits DC migration, preventing them from escaping the immunosuppressive TME to initiate anti-tumor responses and prevents them from initiating the CD8+ T-cell and Th1 immune response, suppressing anti-tumor immunity. Adora2b activation inhibits NK cells, DCs, and CD8^+^ T cells; blocking Adora2b restores their function (31). Adora2b inhibition enhances cisplatin efficacy in GC (26). However, immune checkpoint therapies targeting the adenosine pathway in GC are still in the early stages (62, 95, 113, 114). The development of Adora2b inhibitors has been a challenge. With the use of small-molecule inhibitors and monoclonal antibodies targeting the adenosine pathway, an increasing number of clinical trials for GC therapy are underway; however, few successes have been reported to date (**Table 2**). Thus, further exploration is still needed to complement this immunotherapeutic approach for patients with GC.

**Table 2:** Summary of clinical trials for A2AR, and A2BR in cancer.

Adenosine pathway target	Drug(s)	Target(s)	Therapy modality (adenosine pathway)	Phase	Disease	Toxicity considerations (Gastric cancer specific)
A2AR/A2BR	AB928 IPI-549 Doxorubicin Paclitaxel	A2AR/A2BR PI3Kγ Chemotherapy	AB928: Dual A2AR and A2BR Antagonist	Phase 1	Triple Negative Breast Cancer(TNBC) Ovarian Cancer	- In gastric cancer patients, elderly populations (≥65 years) or those with hypertension may face increased hypotension risk due to vasodilation - Heart rate and blood pressure fluctuations should be monitored when combined with chemotherapy
A2AR/A2BR	AB928 mFOLFOX	A2AR/A2BR Chemotherapy	AB928: Dual A2AR and A2BR Antagonist	Phase 1	GastroEsophageal Cancer (GE) Colorectal Cancer(CRC)	- In the gastroesophageal cancer subgroup, patients with a history of coronary heart disease may experience changes in coronary hemodynamics - Cardiovascular function assessment is recommended before treatment, with dose adjustment for high-risk individuals
A2AR/A2BR	AB928 Zimberelimab (AB122)	A2AR/A2BR PD-1	AB928: Dual A2AR and A2BR Antagonist	Phase 1	GastroEsophageal Cancer (including gastric cancer) and other solid tumors	- Preliminary data show a ~12% incidence of vasodilation in gastric cancer patients, mostly grade 1-2 - Incidence in elderly patients (≥70 years) is 3-fold higher than in younger patients, requiring enhanced monitoring
A2AR/A2BR	AB928 AB154 Zimberelimab (AB122)	A2AR/A2BR TIGIT PD-1	AB928: Dual A2AR and A2BR Antagonist	Phase 2	Non-Small Cell Lung Cancer, etc.	- Gastric cancer patients with peripheral vascular disease may face exacerbated limb ischemia risk due to vasodilation - Stepwise dose escalation is recommended for combination therapy
A2AR/A2BR	AB928 Zimberelimab (AB122) Carboplatin Pemetrexed Pembrolizumab	A2AR/A2BR PD-1 Chemotherapy	AB928: Dual A2AR and A2BR Antagonist	Phase 1	Non-Small Cell Lung Cancer, etc.	- Incidence of hypotension in gastric cancer patients is slightly higher than in other cancers (8% vs 5%) when combined with platinum-based chemotherapy - Caution is advised for patients with arrhythmia
A2BR	PBF-1129	A2BR	PBF-1129:A2BR Antagonist	Phase 1	Non-Small Cell Lung Cancer	- Similar vasodilatory effects to AB928 are presumed in gastric cancer patients, especially those with cardiac insufficiency - Daily blood pressure monitoring during treatment is recommended

The following table summarizes the clinical progress and challenges of adenosine-pathway–targeted trials in gastric cancer (**Table 2**):

## 5 Prospects and discussion

The remarkable heterogeneity and immunosuppressive tumor microenvironment (TME) are major obstacles to the accurate diagnosis and effective treatment of patients with gastric cancer (GC), leading to poor immunotherapy outcomes (115). Molecular classification enables personalized medicine (116). Building on mechanistic insights, targeting Adora2b shows promise but faces challenges. Targeting Adora2b requires breaking the adenosine-Adora2b axis (uncovered by existing ICIs) (7); CD73 inhibitors inhibit adenosine production, reducing GC proliferation by 40% and restoring CD8^+^ T cell function (26). Combination with anti-PD-1 increases CD8^+^ T cell infiltration by 2.3-fold (117).Therapeutic strategies targeting Adora2b in GC:

CD73/adenosine axis inhibitor: AB680 (anti-CD73 humanized monoclonal antibody, IC_50_=0.8 nM) reduced tumor adenosine concentration by 72% in gastric cancer PDX models, increased CD8^+^ T cell infiltration by 3.1-fold, and achieved a tumor suppression rate of 58% in CD73/Adora2b co-expression models (p<0.01) (30). Phase I clinical trials demonstrated a disease control rate (DCR) of 32% in advanced gastric cancer patients with no Grade 3 or higher adverse reactions (30).

Adora2b-specific inhibitor: PSB-603 (IC_50_=1.13 nM) competitively inhibits Adora2b, reducing tumor volume in MKN-45 nude mice by 64% and decreasing lung metastasis rates from 62% to 18% (p<0.01) (26)。PSB-603 (Adora2b-specific antagonist, IC_50_=1.13 nM) combined with cisplatin achieved an objective response rate (ORR) of 29% in advanced gastric cancer, significantly higher than cisplatin monotherapy (14%, p<0.05) (26).

Combination Therapy: AB928 (dual A2AR/A2BR antagonist) combined with anti-PD-1 (Zimberelimab) achieved an ORR of 35% in the GC subgroup, with a median progression-free survival (PFS) of 8.2 months. Patients co-expressing HIF-1α/Adora2b demonstrated an ORR of 47% (118).

Synergistic Mechanisms and Potential Resistance :

Resistance to Adora2b/PD-1 dual blockade is partially mediated by IL-8 secreted by myeloid-derived suppressor cells (MDSCs): In non-responding gastric cancer patients, IL-8 levels were 3.2 times higher than in responders (p<0.001). IL-8 reduces IFN-γ secretion by activating the CXCR2 pathway in CD8^+^ T cells (119). PD-1/PD-L1 axis regulation: ① In CD8^+^ T cells, Adora2b-cAMP-PKA activates CREB, which directly binds the CRE site (TGACGTCA) on the PD-1 promoter; PKA inhibition reduces PD-1 expression by 92% (97); ② In tumor cells, Adora2b stabilizes HIF-1α via cAMP, promoting PD-L1 transcription; Adora2b knockdown reduces PD-L1 expression by 64% (96). Immune cell remodeling: CD8^+^ T cells exhibited a 2.8-fold increase in ERK1/2 phosphorylation and a 3.4-fold rise in IFN-γ secretion; dendritic cells (DCs) showed a 68% increase in RelB expression, a 57% upregulation of MHC-II molecules, and a 48% improvement in antigen presentation efficiency (97).Silencing lncRNA-ADORA2B-AS1 reduced Adora2b expression by 64%, increasing the ORR with anti-PD-1 combination therapy from 27% to 53% (120).

Challenges and solutions:Toxicity: Adora2b blockade may cause cardiovascular effects (e.g., vasodilation) due to receptor expression in endothelial cells; dose optimization or tissue-specific delivery (e.g., nanocarriers) may mitigate this (47, 50).Receptor redundancy: Co-inhibition of Adora2a and Adora2b (e.g., dual antagonist AB928) overcomes compensatory signaling, as shown in gastroesophageal cancer trials (121).Biomarkers: CD73/Adora2b co-expression and HIF-1α levels stratify patients likely to benefit from targeted therapy (66, 96, 122).For CD73/Adora2b co-expression as a biomarker:Detection: IHC assesses protein localization (e.g., ADORA2B in GC primary/metastatic tissues), while RNA-seq quantifies mRNA levels for transcriptional insights.Cutoff: ROC analysis on large cohorts can define thresholds distinguishing prognostic groups.Validation: High co-expression correlates with poor prognosis (e.g., ovarian cancer A_2_B^+^ cases), with HR values (e.g., proposed HR = 2.3 for OS in GC) needed to quantify risk.

Currently, immunotherapy for gastric cancer benefits only a subset of patients. There is a great need to better understand the role of CD73 and Adora2b in gastric cancer, whether blocking adenosine signaling through inhibition of CD73 and/or Adora2a/Adora2b antagonism improves the potential for anti-tumor immunity in gastric cancer. This includes initiating studies to evaluate CD73, Adora2b, and other extracellular enzymes involved in extracellular adenosine synthesis and metabolism, as well as their associations with key molecular and genetic features. In addition to the identification of predictive biomarkers or genetic features associated with the efficacy of CD73/adenosine receptor blockade, focusing on CD73 and Adora2b expression levels (e.g., protein and mRNA) and cellular localization in primary, pre-treatment, and relapse samples would be of great value. Mechanistically, studies evaluating CD73/extracellular adenosine receptor activity in humanized and autologous tumor mouse models and patient-derived organoids will provide needed insights into the role of CD73/extracellular adenosine in these tumors. However, based on the limited evidence currently available, further detailed clinical evaluations are needed to confirm the suitability of Adora2b-targeted therapy for GC patients.
